# Single-cell and spatial analyses of cancer cells: toward elucidating the molecular mechanisms of clonal evolution and drug resistance acquisition

**DOI:** 10.1186/s41232-021-00170-x

**Published:** 2021-07-16

**Authors:** Satoi Nagasawa, Yukie Kashima, Ayako Suzuki, Yutaka Suzuki

**Affiliations:** Department of Computational Biology and Medical Sciences, Graduate School of Frontier Sciences, The University of Tokyo, 5-1-5, Kashiwanoha, Kashiwa-shi, Chiba, 277-8561 Japan

**Keywords:** Single-cell RNA-seq, Anticancer drug resistance, Single-cell multiome analysis, Spatial transcriptome analysis

## Abstract

Even within a single type of cancer, cells of various types exist and play interrelated roles. Each of the individual cells resides in a distinct microenvironment and behaves differently. Such heterogeneity is the most cumbersome nature of cancers, which is occasionally uncountable when effective prevention or total elimination of cancers is attempted. To understand the heterogeneous nature of each cell, the use of conventional methods for the analysis of “bulk” cells is insufficient. Although some methods are high-throughput and compressive regarding the genes being detected, the obtained data would be from the cell mass, and the average of a large number of the component cells would no longer be measured. Single-cell analysis, which has developed rapidly in recent years, is causing a drastic change. Genome, transcriptome, and epigenome analyses at single-cell resolution currently target cancer cells, cancer-associated fibroblasts, endothelial cells of vessels, and circulating and infiltrating immune cells. In fact, surprisingly diverse features of clonal evolution of cancer cells, during the development of cancer or acquisition of drug resistance, accompanied by corresponding gene expression changes in the circumstantial stromal cells, appeared in recent single-cell analyses. Based on the obtained novel insights, better optimal drug selection and new drug administration sequences were started. Even a remaining concern of the single cell analyses is being addressed. Until very recently, it was impossible to obtain positional information of cells in cancer via single-cell analysis because such information is lost during preparation of single-cell suspensions. A new method, collectively called spatial transcriptome (ST) analysis, has been developed and rapidly applied to various clinical specimens. In this review, we first outline the recent achievements of single-cell cancer analysis in analyzing the molecular basis underlying the acquisition of drug resistance, particularly focusing on the latest anti-epidermal growth factor receptor tyrosine kinase inhibitor, osimertinib. Further, we review the currently available ST analysis methods and introduce our recent attempts regarding the respective topics.

## Background

A detailed understanding of cellular diversity and constituting ecosystems of cancers is expected to serve as a foundation for cancer eradication and control. Particularly, heterogeneity of cancer cells derived from clonal evolution during anticancer drug treatment is directly related to drug resistance and thus is crucial for treatment success. The selection of the optimal drug and drug administration procedure is not always based on molecular evidence, considering the possible emergence of drug-resistant cells. To this end, conventional omics analysis approaches such as RNA-seq, chromatin immunoprecipitation sequencing (ChIP-seq), and whole-genome/exome sequencing (WGS/WES) have limited power, although they may be comprehensive regarding the covered genes and genome. These methods usually use the “bulk” for the material. Inherently, they measure the average value of a large number of component cells; therefore, they cannot detect the behavior of minor cells or the presence of heterogeneous cellular populations therein. For this purpose, an individual cell analysis, so-called single-cell analysis, is needed. Indeed, several papers have been reported. For example, Kim et al. combined single-nucleus RNA-seq, single-nucleus DNA-seq (for copy number profiling), and bulk exome sequencing [1].

Despite the substantial advantages of single-cell analysis, the current single-cell analytical methods have been argued against due to several drawbacks. The most significant drawback is that the positional information of various cells is lost when single cells are dissociated from the tissue. With this disadvantage, it is sometimes difficult to grasp the whole picture of the interconnected structure of the precancerous, cancer, drug-resistant, and transient cells of those stages. Spatial transcriptome (ST) analysis was recently developed to address this flaw. Here, we review recent single-cell analysis approaches for a better understanding of cancer heterogeneity. A particular focus will be placed on the molecular mechanisms underlying drug resistance. Additionally, we introduce ongoing ST analysis-based studies aimed at further exploring the spatial heterogeneity of cancer cells. Finally, perspectives on the integration of these two approaches are discussed.

## Main text

### Single-cell sequencing for intratumor heterogeneity of cancer cells

#### Single-cell analysis for understanding intratumor heterogeneity and clonal evolution of cancer cells

During cancer progression, tumor cells proliferate with an accumulation of genomic mutations. Single-cell genome sequencing is potentially the most direct method for analyzing intratumor heterogeneity and cancer evolution. However, it is practically impossible to obtain precise single-cell genome data simply because an individual human cell has only two DNA copies. In addition, substantial sequencing depth is needed to cover the entire human genome. Single-cell copy number analysis has been employed in several studies to elucidate cancer genomic diversity instead of in-depth whole genome sequencing. From relatively shallow genomic sequence data, an evolutionary model for each critical phase of cancer, including clonal proliferation, invasion, relapse, and metastasis, was successfully constructed [2–5]. It was also shown to be possible to estimate copy number aberrations and clonal structure from single-cell RNA sequencing (scRNA-seq) data, based on the assumption that largescale chromosomal aberrations would affect the transcriptome features of the genes residing therein. Several computational methods, such as inferCNV [6] and CopyKAT [7], have been developed for this purpose.

Furthermore, epigenomic and transcriptomic features change during cancer progression, providing a gene expression base for malignant phenotypes, such as dedifferentiation and epithelial-mesenchymal transition (EMT). scRNA-seq analysis is the most powerful tool for inferring the transition statuses of cancers. For this purpose, a computational method called pseudo-time analysis was employed. In glioblastoma, for example, transcriptomic heterogeneity and their transition during the differentiation of cancers have been analyzed. In 2014, Patel et al. reported scRNA-seq analysis using 430 primary glioblastomas cells. They classified cells at various stages as cells having stem-like to fully differentiated signatures [6]. In recent studies, a much larger number of cells have been analyzed. These studies have collectively delineated a complicated lineage hierarchy and differentiation paths of glioblastoma cells and their transcriptional regulators [8–10].

#### Single-cell analysis for characterizing microenvironmental non-tumor cells

There have been a large number of single-cell studies focusing on non-tumor cells and their microenvironment. The first study of such an approach, designated as a “cancer ecosystem” study, was conducted for metastatic melanoma [11], in which 4645 cells, including cancer cells and non-malignant cells, were analyzed. Another study conducted by Lambrechts et al., for example, reported a single-cell transcriptome catalog of stromal cells in lung cancer patients [12]. They analyzed approximately 100,000 cells (52,698 cells for the initial catalog and 40,250 cells for validation) and identified 52 stromal cell clusters, including immune cells, endothelial cells, and fibroblasts. They characterized each type of stromal cell and found that their distribution was associated with tumor characteristics and patient prognosis.

**Fig. 1:**
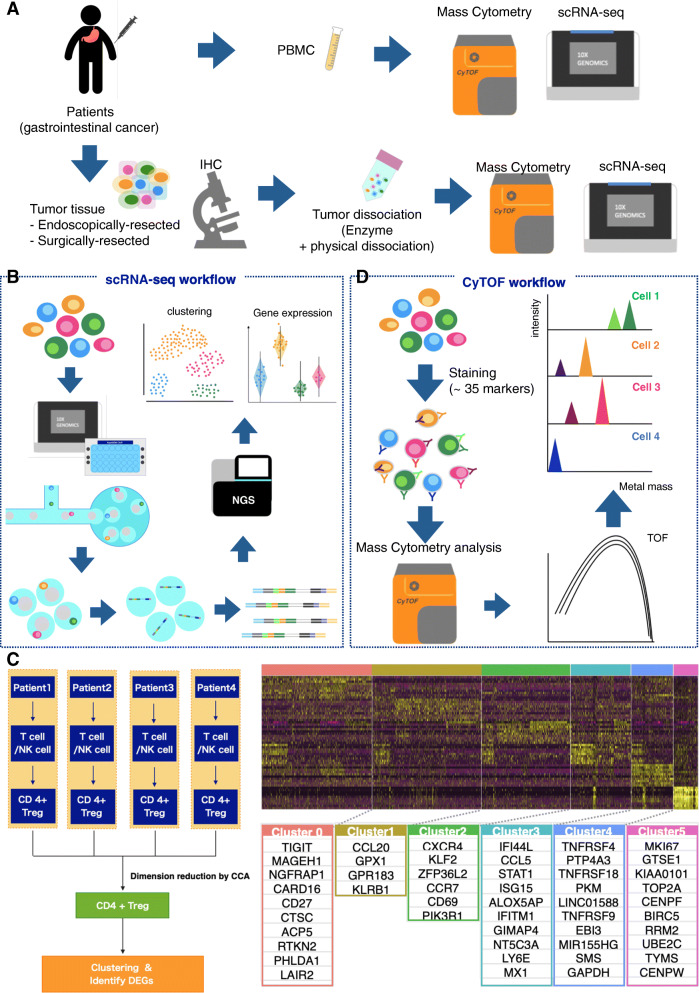
Scheme of the study using scRNA-seq, CyTOF, and IHC. To demonstrate the potential of the integrated analysis, we analyzed PBMCs and endoscopically and surgically resected samples from patients with gastrointestinal cancer (**a**). We used microdroplet-based single-cell system, 10x Genomics Chromium, for scRNA-seq analysis (**b**). We focused on regulatory T cell and revealed subgroup of them (**c**). In addition, we conducted mass cytometry using CyTOF Fluidigm (**d**)

In recent years, tumor-infiltrating lymphocytes (TILs) have received much attention. Their omics features might be associated with patient prognosis and efficacy of immunotherapies, such as immune checkpoint inhibitors (ICIs) [13]. Numerous studies have appeared to be related to TILs, such as CD8^+^ T cells, regulatory T cells, dendritic cells, and tumor-associated macrophages (TAMs). For example, a research group at Stanford University reported immune cell profiling using scRNA-seq before and after anti-PD-1 treatment for basal cell carcinoma patients [14]. They found that pre-existing TILs were neither activated nor expanded after PD-1 blockade; instead, novel T cell clones appeared to undergo sequential reactions. GAPFREE2 consortium, including the authors of this manuscript, also conducted scRNA-seq using clinical samples from gastrointestinal cancer patients to characterize the transcriptome features of a series of cells constructing cancers, primarily focusing on immune cells [15] (Fig. 1a). In this study, we employed 10x Genomics Chromium scRNA-seq system, which is based on the micro-droplet system [16]. Briefly, cells resuspended in reagent for reverse transcription, Gel beads for barcoding and oil for making droplet are loaded in the chip. After the reverse transcription, each cell has unique cell barcode, 10x BC, and each mRNA has unique molecular identifier, UMI, thus we can process pooled samples and make library for sequencing. With those 10xBC and UMI, we can identify expression level of each cell (Fig. 1b). We characterized several groups of regulatory T cells that infiltrated cancer tissues using scRNA-seq (Fig. 1c). In this study, we also performed protein level analysis using cytometry by time of flight (CyTOF), as well as immunohistochemistry (IHC) concurrently with scRNA-seq. Basically, mass cytometry, including CyTOF, is a combination of flow cytometry and elemental mass spectrometry. It enabled us to analyze cell with much more parameters in the quantitative way. In CyTOF analysis, cells are stained with markers simultaneously. Though FACS analysis use fluorescence, CyTOF use heavy metal tags [17]. After staining, cells are processed in the machine (Fig. 1d). We compared immune cell compositions detected via scRNA-seq and CyTOF using PBMCs and biopsy/surgically dissected cancer tissues. Some unexpected cells, such as plasma cells, could only be characterized in scRNA-seq datasets because CyTOF could only measure the levels of the preset markers. In contrast, CyTOF is known to have advantage when we have specific target for analysis [17]. It could more clearly classify some cell types, such as NK cells, due to differences in the expression of cell-type markers at the mRNA and protein levels. Their results demonstrated that scRNA-seq could be an even more powerful tool when used in combination with CyTOF by mutually complementing weak points [17, 18]. We summarize the character of scRNA-seq and CyTOF in Table 1.

**Table 1:** Comparison of instrument shown in this paper

	scRNA-seq (10x Genomics)	CyTOF (Fluidigm)	scATAC-seq (10xGenomics)	scGEX-ATAC (10xGenomics)	Visium
Basic information	Single-cell RNA sequencing	Cytometry by Time-Of-Flight (elemental mass spectometry + flow cytometry)	Single-cell assay for transposase-accessible chromatin	Single-cell RNA-seq and ATAC-seq	Spatial Transcriptome
Required input	870–17,400 cell (after wash/5'GEX)	1,000,000–3,000,000 cells	775–15,400 nuclei (after wash)	775–15,400 nuclei (after wash)	
Expected output	500–10,000 cells	~ 200,000	500–10,000 cells	500–10,000 nuclei	Up to 5000 data spot
Number of markers can be analyzed	~ 500–~ 5000 (depend on sample type)	30–40 proteins	~ 500–~ 5000 (depend on sample type)	depend on sample type	Transcriptome
Tool for infomatic analysis	Cell Ranger, R package	Cytobank, Pathsetter, R packages	Cell Ranger, R packages	Cell Ranger ARC, R packages	Cell Ranger, R packages
Pros	・Reveal whole transcriptome dataset as long as the gene has enough expression level	・Reveal expression of targeted protein levels of each cell ・Low cost compare to scRNA-seq and scATAC-seq ・Identify > 30 immune cell type easily (pre-set) ・Have advantage in highly-target cell analysis	・Reveal cell heterogeneity in chromatin level	・Reveal cell heterogeneity in transcriptome and chromatin level simultaneously	・Gene expression can be obtained while maintaining spatial information.
Cons	・Low cell number of output compared to CyTOF ・Low depth reads compared to conventional bulk RNA-seq ・Unable to use cell after processing	・Can use only pre-determined markers ・Unable to use cell after processing ・Affected by sensitivity of ion used in the staining	・Difficult to get comprehensive coverage of open chromatin sites ・Unable to use cell after processing	・Cannot detect transcriptome use nuclei, not cell cytoplasm	・At this stage, the resolution is not at the level of a single cell

### Single-cell sequencing unveils molecular mechanisms of anticancer drug resistance

Anticancer drugs are used for surgically inoperable cancer tissues that have already developed metastasis, severe invasion, or recurrence. Molecular targeting drugs that target signaling molecules specifically expressed in cancer cells have been administered in clinical practice in addition to traditional cytotoxic anticancer drugs, leading to improved clinical outcomes. However, there are still not a few cases in which the cancers become drug-resistant and recur after a certain period of post-drug treatment. One of the most significant barriers to the complete elimination of cancer is the emergence of drug-resistant cells during cancer evolution with a complicated microenvironment in cancer tissues. Several important studies have been performed to elucidate the drug resistance mechanism of cancer cells using single-cell sequencing technologies and have revealed the mechanisms of resistance to anticancer drugs, such as molecular targeting drugs, conventional cytotoxic drugs, and ICIs.

#### Single-cell sequencing presented the hypothesis for mechanisms of drug resistance

In 2018, Ho et al. analyzed the mechanism of BRAF inhibitors in melanoma using single-cell RNA-seq analysis and K/clustering evaluation (SAKE), which can process the data analysis from QC to enrichment analysis and provide detailed gene expression profiles. They compared SAKE with known scRNA-seq data analysis tools, SC3, Seurat, SINCERA, CIDR, and RaceID, and demonstrated its accuracy and robustness. They applied SAKE to reveal the mechanism of resistance to BRAF inhibitors using the Fluidigm C1 system, 10x Genomics Chromium, and bulk RNA-seq and indicated that a rare population of resistant cells to BRAF-i exists ab initio [19].

Sharma et al. focused on the resistance mechanism of both phenotypically homogeneous and heterogeneous cell lineages using patient-derived primary oral squamous cell carcinoma (OSCC) cell lines. They showed that cells with high heterogeneity have an advantage in the selection of intratumor heterogeneity. In contrast, cells with high homogeneity utilize tumor evolution via epigenetic changes, and the stem cell factors *SOX2* and *SOX9* play a role in this drug-induced adaptation [20].

Kim et al. conducted single-cell DNA/RNA analysis of breast cancer specimens before and after chemotherapy to elucidate that cancer cell heterogeneity is a cause of drug tolerance. Samples from 20 triple-negative breast cancer (TNBC) patients undergoing neoadjuvant chemotherapy (NAC) showed pre-existing and drug-induced selection of NAC-resistant tumor cells [1]. Schnepp et al. also focused on chemotherapy resistance; using two prostate cancer cell line models, a docetaxel-sensitive variant and a docetaxel-resistant variant, they showed that NP1 was a key regulator [21].

Jerby-Arnon et al. reported that there are multiple mechanisms of resistance to an ICI. They conducted scRNA-seq and bulk RNA-seq of 33 melanoma tumors. Based on the results, they demonstrated that their predictive biomarkers and combination therapy are effective [22].

Collectively, it has been revealed that both of the following mechanisms are possible and have been observed: (i) the primary mechanism to acquire drug resistance is a series of genomic mutations and their clonal expansion at the same time as the clonal evolution for further fitting the niche; (ii) the tumor-forming cancer cells have a certain diversity, originally including already drug-resistant cells; and (iii) gene expression was reprogrammed after responding to chemotherapy and changed to a newly acquired expression profile.

#### Revealing the mechanism of resistance to epidermal growth factor receptor tyrosine kinase inhibitors using the single-cell technology

**Fig. 2:**
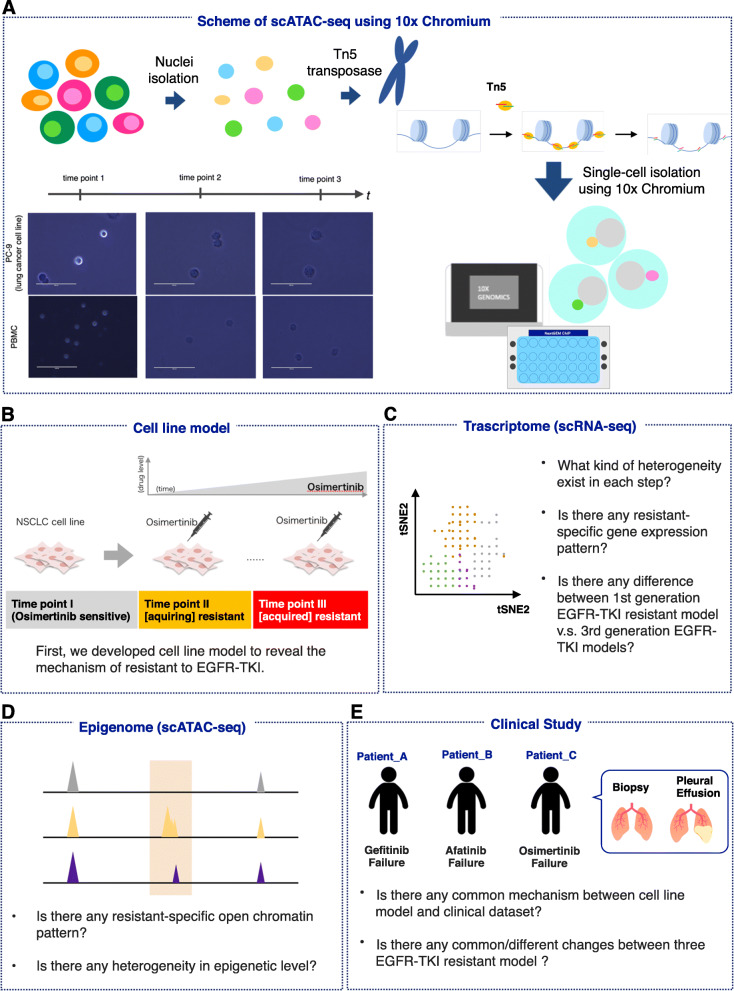
Scheme of the study revealing the EGFR-TKI resistance mechanism. For scATAC-seq, we used 10x Genomics. Before barcoding, nuclei were isolated and processed with Tn5 transpose. The processing time for nuclei isolation was determined using microscope (**a**). To reveal the resistance mechanism, we first established an EGFR-TKI-resistant cell line (**b**). We then analyzed this cell line model using scRNA-seq (**c**) and scATAC-seq (**d**). Additionally, we conducted scRNA-seq with clinical samples to confirm that the candidate mechanism occurs in vitro and in vivo (**e**)

Our study focused on the mechanism of resistance acquisition to epidermal growth factor receptor tyrosine kinase inhibitors (EGFR-TKIs) in non-small cell lung cancers (NSCLCs). Resistance to EGFR-TKIs in tumors is known to be complex. The most well-elucidated somatic mutations in the *EGFR* genes are detected in 10–30% of NSCLC cases. These mutations promote downstream pro-survival and anti-apoptotic signals [23, 24]. Although most patients with *EGFR* mutations show dramatic responses to first- and second-generation EGFR-TKIs, such as gefitinib and erlotinib, and afatinib and dacomitinib, respectively, the majority of patients subsequently acquire resistance within 2 years [25]. Osimertinib, a third-generation EGFR-TKI, was recently developed to target cells harboring a T790M resistance mutation. However, even for osimertinib, the emergence of resistance mutations has already been reported [26, 27]. We considered that resistance acquisition, which may occur in a small population of cells, can be identified by single-cell multiomics analysis. Our group, in collaboration with Kobayashi group at National Cancer Center of Japan, attempted to identify key gene expression changes during the process of resistance acquisition, first using cell lines and then clinical specimens through scRNA-seq and single-cell assay for transposase-accessible chromatin using sequencing (scATAC-seq). ATAC-seq, assay for transposase-accessible chromatin using sequencing, was first reported in 2013. Using ATAC-seq, we can know regulatory region by inserting sequence adaptors into accessible region of the genomes. One of the advances of ATAC-seq is it requires only low sample input. Single-cell ATAC-seq was reported in 2015 [28, 29]. First, microfluidic based system was used for scATAC-seq, thus it can process only limited number of cells. However, currently, we can use 10x Genomics for scATAC-seq and analyze much more number of cells [30]. In scATAC-seq using 10x Genomics, cells are processed to isolate nuclei. The condition for nuclei isolation depends on the sample conditions. We show examples of processing of PBMC and cell line (Fig. 2a, left bottom). After nuclei isolations, samples are processed with transposase Tn5. Next, samples are encapsulated with beads with barcode using Chromium. We make libraries for sequencing. Detailed information for ATAC-seq is described in Buenrosto et al. [31]. We also summarized the character of scATAC-seq in Table 1 [32]. Schematic representation of the study design is shown in Fig. 2b–e. Collectively, we believe molecular features at multiple omics layers at single cell resolution would provide new insights into the mechanisms of EGFR-TKIs resistance (Kashima et al. submitted).

### Overcoming the limitation of recent single-cell technologies: single-cell multiome analysis

**Fig. 3:**
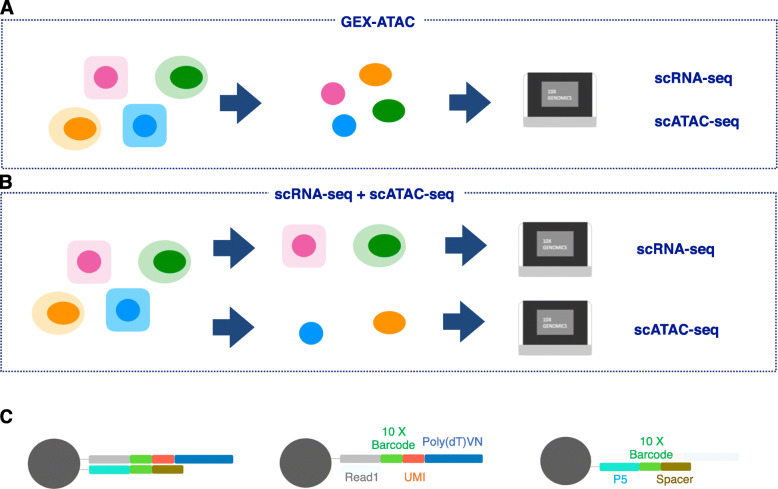
Overview of previous and current single-cell multiome analysis. In the previous version of the 10x chromium system, we could not use identical cells in both scRNA-seq and scATAC-seq (**a**). We acquired the dataset from an identical cell using the new system (**b**). Due to changes in sample preparation before barcoding, nuclear RNA, not cellular RNA, was analyzed in the new system. **c** Each gel bead has two types of primers to identify each cell (left): primers for scRNA-seq (middle) and primers for scATAC-seq (right) (**c**). Using 10x barcodes, we could distinguish cells in both the scATAC-seq dataset and the scRNA-seq dataset

Until very recently, for the scRNA-seq and scATAC-seq analyses, the sample was prepared independently due to technical limitations. Therefore, it was impossible to associate epigenomic and transcriptomic features for each single cell directly. Recently, a novel method called GEX-ATAC sequencing has been developed. In this method, it is possible to conduct both scRNA-seq and scATAC-seq simultaneously for a given single cell. The representative commercial kit was released from 10x Genomics. Although most of the workflow for library preparation is similar to that of previous scRNA-seq or scATAC seq, GEX-ATAC employs several unique processes. First, GEX-ATAC utilizes nuclei as a material (Fig. 3a). Therefore, the transcriptome data represented by GEX-ATAC is from nuclear mRNA; thus, it is not always consistent with cytosolic mRNA (Fig. 3b). Second, as shown in Fig. 3c, each gel bead has two types of primers: one is a primer for scATAC-seq, and the other is for scRNA-seq (Fig. 3c, left). The primer for scRNA-seq consisted of Illumina Truseq Read1, 16 nt of 10x Barcodes, working as a cell identifier, unique molecular identifier (UMI), and poly(dT)VN (Fig. 3c, middle). The other primer for scATAC-seq consisted of the Illumina P5 sequence, 16 nt of 10x Barcodes, and spacer (Fig. 3c, right). After library construction and sequencing, the sequence data were separated into scRNA-seq or scATAC-seq and each individual cell based on these primer sequences (10x Genomics User guide CG000338 RevA). A technical advantage of GEX-ATAC is that it can be started with frozen samples, which may broaden the sample selection subjected to the analysis (10x Genomics website: https://www.10xgenomics.com/products/single-cell-multiome-atac-plus-gene-expression).

### Overcoming the limitation of the recent single-cell technologies: ST analysis

As described above, in a substantial number of cases for various cancer species, analyses have been performed to characterize the diverse behavior of individual cells in a given cancer. However, it has been argued that a novel approach is needed to quantify single cells that retain pathological spatial information. The field of ST analysis has emerged to address this issue. Recently, this field has expanded rapidly, and new methods have been developed one after another. These large numbers of methodologies have fundamentally different approaches, with advantages and disadvantages unique to each method and many issues to be resolved, such as dependence on sensitivity, effort, and organizational type. None of the single methods can use all of these important parameters. In this chapter, we outline the available ST analysis techniques in four groups according to the differences in their approaches: (1) microdissection-based, (2) in situ hybridization-based (ISH), (3) in situ sequencing-based (ISS), and (4) in situ capture-based (ISC). Table 1 presents a comparison of the methods. Further, we present an example of ST analysis using Visium from 10x Genomics, a commercial version of ISC-based ST analysis.

#### Microdissection-based approach

The most robust method for obtaining spatial gene expression information is to perform gene expression profiling of the targeted areas visually specified from the tissues. In laser-capture microdissection (LCM), after physically excising the target area with a laser beam under a microscope, RNA is extracted in vitro, and the gene expression information is acquired [33, 34]. In 2017, Chen et al. introduced an extended version of the LCM protocol named geographic position sequencing (Geo-Seq) that combines LCM and scRNA-seq technology [35]. They obtained the transcriptome of a small tissue region equivalent to 10 cells while maintaining positional information. While it is possible to completely extract the different types of RNA by resectioning individual regions, such as analyzing feasible isoforms, the throughput is limited due to the time and effort of the experimental procedure. It is difficult to grasp the entire image of the tissue because the analysis area is quite limited.

A method that physically resects and acquires localized spatial gene expression data and LCM thinly slices tissue samples and prepares individual sequencing libraries from each slice. In 2014, RNA tomography (tomo-seq) was performed, in which zebrafish embryos were sliced along the body axis at a thickness of 18 μm, and RNA was extracted from each frozen section (~ 100 sections) to obtain gene expression information. They then computationally reconstructed the 3D transcriptional profile of the embryo by overlapping RNA-seq information from all frozen sections [36].

The transcriptome in vivo analysis (TIVA) was published in 2014 to perform ST analysis of any region using photonics [37]. This method introduces a light-activated polyA oligo called a TIVA-tag into living cells in the tissue. This oligo has a cell transmembrane peptide that allows it to enter the cytoplasm. By irradiating the laser beam to any targeted cell, the TIVA-tag is activated inside the cell and hybridizes with the cellular mRNA. Thereafter, the TIVA-tag-mRNA hybrid was purified from the selected cells with streptavidin, and the captured mRNA was analyzed by RNA-seq. Although it is an excellent method using optical engineering to achieve single-cell resolution, it is currently limited to living cells.

In 2017, Medaglia et al. developed NICHE-seq as a method that used photoactivation to determine the cellular and molecular composition of the microenvironment and demonstrated the experiment using mice expressing photoactivatable GFP [38]. In this method, it is possible to analyze thousands of cells in a microenvironment, but the exact location of the cells in the photoactivated region remains unknown. In 2018, Boisset et al. reported ProximID focusing on the actual physical interaction between cells to construct cellular networks, although the location of cells in the microenvironment is still unknown [39]. This method creates a cell structural unit containing two or three interacting cells by dissociating the tissue under mild conditions so that the small interaction structure is maintained. Then, by performing scRNA-seq on cell structural units collected manually, it shows that we can find a new cell-cell interaction even if we do not know the constituent cell types in advance. However, the location information at the tissue level is unknown because of the low throughput due to manual work and the small amount of cell masse.

#### ISH-based approach

In medicine and molecular biology, the term “in situ” refers to “the original place in the organism where the cells belong.” Instead of the abovementioned approach of resecting individual cells (or parts) in the tissue and extracting RNA molecules, ISH-based methods are available by directly visualizing RNA where it should be. ISH uses a probe complementary to the nucleic acid and hybridizes it in a cell. This technology has existed since the 1960s and has been used to visualize gene expression since the late 1970s [40]. The single-molecule RNA fluorescence in situ hybridization (smFISH) method has achieved high sensitivity by hybridizing multiple short fluorescent-labeled probes to different regions of the transcript [41]. It is still of general use because of its high intracellular spatial resolution and the feasibility of quantitative measurement of transcripts. The intracellular location of RNA molecules can provide important information on biological implications as a snapshot of transcription. However, only a few genes can be targeted at once because of the limit of spectral overlapping in standard microscopy. Cai et al. reported the seqFISH method in 2014 [42]. This method involves hybridization of the fluorescent-labeled probes (single-stranded DNA) to intracellular RNA and imaging, after which the probes are stripped with DNase I and hybridized again with different fluorescent-labeled probes. By repeating this cycle, the mRNA-type information is converted into color information, and the gene is identified by the combination pattern. Theoretically, by repeating N times with F types of fluorescent dyes, it is possible to identify the RNA types of F to the Nth power. However, in practice, errors and noise are introduced from the measurements, such as gaps in positional information, when repeating measurements. In 2015, Zhuang et al. identified 1001 genes using the multiplexed error-robust FISH method. The imaging algorithm and probe design were optimized to correct the errors and restore the true signal from the data, including errors [43]. In 2019, Cai et al. reported that seqFISH successfully identified approximately 10,000 genes by adopting a similar concept (seqFISH+) [44]. However, the throughput and adaptation to large tissues are concerns because both methods require optical resolution at the cellular level for imaging. When simultaneously detecting a larger number of different transcripts, ISH-based methods requiring a known target sequence had significant overlapping spectrum restrictions. With recent advancements in ingenious sequencing strategies, it is possible to obtain almost all transcriptome readouts.

#### ISS-based

In the ISH-based methods described above, the spatial gene expression in tissues or cells was detected by barcoding the fluorescent materials and probes to “see” the expressed genes in the cells. In contrast, ISS-based methods attempt to detect nucleotide sequences by directly “reading” them. In 2013, the first ISS approach using a padlock probe (PLP) targeting a known gene was reported [45, 46]. This method first reverse-transcribed mRNA in the tissue to synthesize cDNA. Hybridizing PLPs, micrometer-sized RCA products (RCPs) are obtained by a targeted amplification method called rolling circle amplification (RCA). The genes were then identified by decoding the RCP nucleotide sequence using the sequencing-by-ligation (SBL) method. In 2017, CARTANA was established to commercialize padlock-based ISS technology, with commercialization planned by 10x Genomics. In 2020, the HybISS method was presented as an improved version of the padlock-based ISS technology, with SBL changing to sequencing-by-hybridization (SBH) [47]. They modified the probe design, which allows for a new barcoding system via SBH chemistry for an increased signal-to-noise ratio. In a method called barcode in situ targeted sequencing (BaristaSeq) reported in 2018, the read length was extended by cross-linking RCPs with extracellular matrices and using the sequencing-by-synthesis (SBS) method used in Illumina sequencers for base decoding to achieve longer read lengths, up to 15 bases [48]. STARmap (spatially resolved transcript amplicon readout mapping) reported by Stanford University in 2018 is another approach using PLP [45, 46]. In this method, it is possible to bypass the reverse transcription step by using a set of primers and PLPs that hybridize to a specific RNA, called NSAIL. In the following step, the RCPs are amplified using the RCA method. Incorporated by the amine-modified bases during RCA, the RCPs were fixed in the location. After removing proteins and lipids to increase tissue permeability, sequencing was performed using the modified SBL method. The PLPs contain a 5-mer barcode that labels each gene, and the amplified DNA can be identified by the sequence of the 5-mer barcode depending on the gene. The possible analysis in 3D is the most distinctive point of this method. According to the paper, the resolution is still 100–150 μm thick, but it is expected to be further improved by increasing the 5-mer of the barcode. The methods described above are based on known sequences. In 2014, a method named fluorescent in situ RNA sequencing (FISSEQ) was reported, which enables the capture of unspecified RNAs [49]. This method involves RNA reverse transcription using random primers. Incorporation of aminoallyl dUTPs makes it possible to cross-link aminoallyl dUTPs with BS (PEG)9 and allow RCPs to be anchored to the cell protein substrate. Finally, the SBL method determines the sequences of reading lengths of 30 bases.

#### ISC-based approach

Although ISH and ISS enabled the identification of molecules at the cellular level resolution, they require large systems. In contrast, ISC-based methods, an approach that conducts ex-situ sequencing after capturing RNA in situ, may facilitate gene analysis while maintaining the spatial organization of the tissue relatively easily.

The spatial transcriptomics technology was developed in 2016 at the Karolinska Institute, Sweden, as the first technology to adopt this approach [50]. In this method, the oligo dT primer to trap poly A-RNA was pre-coated onto the glass slides on which the tissue sections were attached. Each primer was preloaded with a barcode to identify the location of the coordinates on the glass slide. Specifically, the slide area was subdivided into spots with a diameter of 100 μm, with different barcode-labeled capture primer sticks on each spot. Upon attaching the tissue to a slide and disrupting the cell membrane, mRNA in the tissue was captured by nearby primers. Adding reverse transcriptase to synthesize cDNA makes it possible to synthesize a gene library that reflects its localization. Sequences were determined by next-generation sequencing. Because the determined sequence contains the coordinate information, the detailed expression information of the coordinates can be obtained. The 10x Genomics company acquired, developed, and improved this ST technology to 55 μm/diameter resolution and commercialized it under the name “10x Visium” at the end of 2018. Instead of using oligos, barcoded beads named pucks were laid on glass slides by a method called slide-seq to achieve a concept similar to the ST technique by a group at the Massachusetts Institute of Technology [51]. Because the beads have a diameter of 10 μm and a resolution higher than that of the STs described above, it is possible to acquire information at the single-cell level. However, unlike ST, it should be noted that the current version has limited sensitivity and needs the support of scRNA-seq data for proper cell-type mapping. In addition, tissue images can be obtained from adjacent sections but not from the same sections that provide RNA data. Shortly after presenting slide-seq, a method using smaller barcode beads named high-definition spatial transcriptomics (HDST) was reported by the group that developed ST [52]. This bead with a diameter of 2 μm has a higher resolution, but the proper mapping of cell types still requires supporting scRNA-seq data. One method that can be adapted to formalin-fixed paraffin-embedded (FFPE) samples is digital spatial profiling (DSP) [53]. In this method, FFPE samples are stained with indexing oligonucleotides covalently attached to primary antibodies or mRNA hybridization probes with a UV-photocleavable (PC) linker. The user manually selects regions of interest (ROIs) followed by excitation with UV light. UV cleaves oligo-off antibodies in the ROI. Photocleaved oligos are collected and counted digitally using nCounter or a next-generation sequencer readout. A comparison of each ST seq technology is shown in Table 2.

**Table 2:** Comparison of ST seq technologies

Method name	Year	Resolution	Sample	Information	Pros/Cons
Microdissection-based					
LCM [33, 34]	1996	ROI	FF/FFPE	Transcriptome/Target	Robust/Low throughput
tomo-seq [36]	2014	3D tissue	FF	Transcriptome	3D constructable/Multiple identical biological samples are required
TIVA [37]	2014	single cell	Fresh cells	Transcriptome	Can be applied to live cells/Low throughput
Geo-seq [35]	2017	10 cells	FF	Transcriptome	Extended version of the LCM/Low throughput
NICHE-seq [38]	2017	ROI	Fresh cells	Transcriptome	Can be applied to live cells/Lost spatial information within the region of interest
ProximID [39]	2018	2–3 cells	Fresh cells	Transcriptome	Reflects the physical relationship between cells/Low throughput
In situ hybridization-based					
RNA-ISH [40]	late 1970	Subcellular	Ethanole fixes	gene	High sensitivity/Low throughput
smFISH [41]	2008	Subcellular	FF/FFPE	Target (some gene)	High sensitivity/Low throughput
seqFISH [42]	2014	Subcellular	FF	Target (12gene)	Multiplex/Small observation area, not single molecular
MERFISH [43]	2015	Subcellular	FF	Target (1001gene)	Multiplex/Small observation area, not single molecular
seqFISH+ [44]	2019	Subcellular	FF	Target (10000gene)	Highly multiplex, resolution/Small observation area, High cost
In situ sequencing-based					
padlock-based ISS technology [46]	2013	Subcellular	FF/FFPE	Target (~ 100gene)	Can detect SNVs/limited gene number
FISSEQ [49]	2014	Subcellular	FF/FFPE	Transcriptome	Non target/Small observation area
BaristaSeq [48]	2018	Subcellular	cell line	Target	Can detect SNVs/Target sequence is necessary
STARmap [45]	2018	Subcellular	Fresh cell/FF	Target (1024gene)	Avoiding RTstep, 3D constructable/Small observation area
HybISS [47]	2020	Subcellular	FF	Target	Improved version of the padlock-based ISS technology/Small observation area
In situ capturing-based					
Spatial Transcriptome [50]	2016	20–100 cells	FF	Transcriptome	Whole transcriptome, Wide observation area/not single cell resolution
Slide-seq [51]	2019	Single cell (10um)	FF	Transcriptome	High resolution/Low sensitivity
HDST [52]	2019	Single cell (2um)	FF	Transcriptome	High resolution/Low sensitivity
Digital Spatial Profiling [53]	2020	ROI (1-5000cell)	FFPE	Target (~ 5000 gene)	Compatible with FFPE/Small observation area

*LCM* laser capture microdissection, *ROI* region of interest, *FF* fresh frozen, *FFPE* formalin fixed paraffin embedded

### An example of STseq applications

**Fig. 4:**
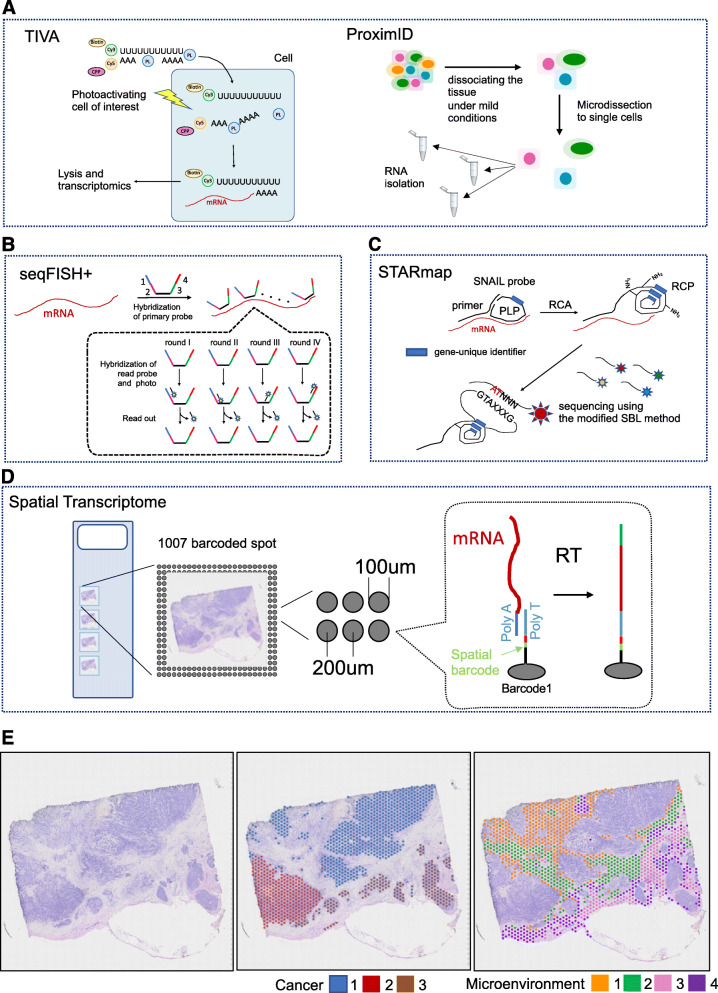
Overview of each ST seq technologies and Spatial transcriptome analysis of breast cancer using Visium. Overview of typical technologies based on microdissection (**a**). TIVA; this method introduces a photo-activated polyA oligo called a TIVA-tag into living cells in the tissue. This oligo has a cell penetrating peptide (CPP) that allows it to enter the cytoplasm. By irradiating the laser beam to cells of interest, the TIVA-tag is activated inside the cell and hybridizes with the cellular mRNA. The TIVA-tag-mRNA hybrid was purified from the selected cells with streptavidin, and the captured mRNA was analyzed by RNA-seq. ProximID; this method creates a cell structural unit containing two or three interacting cells by dissociating the tissue under mild conditions. Then, by performing scRNA-seq on cell structural units collected manually, it shows that we can find a new cell-cell interaction even if we do not know the constituent cell types in advance. Overview of typical technologies based on in situ hybridization (**b**). seqFISH; in this method, a primary probe incorporating four read probes (1–4) is hybridized per arbitrary mRNA. This expansion of the read sequence has dramatically increased the number of genes that can be analyzed. As in smFISH, fluorescently labeled read probes (indicated by a star) are bound to the read sequences and photographed for detection. After that, this reading probe is stripped, a new reading probe is hybridized, and imaging is performed. By repeating this operation 80 times per fluorescence, theoretically 24,000 genes can be detected. Overview of typical technologies based on in situ sequencing (**c**). STARmap; in this ISS method, it is possible to bypass the reverse transcription step by using a set of primers and PLPs that hybridize to a specific RNA, called SNAIL (Specific Amplification of Nucleic Acids via Intramolecular Ligation). The RCPs are amplified using the RCA method. After removing proteins and lipids to increase tissue permeability, sequencing was performed using the modified SBL method. Overview of typical technologies based on in situ capturing (**d**). Spatial Transcriptome; in this method, the oligo dT primer to trap poly A-RNA was pre-coated onto the glass slides on which the tissue sections were attached. Each primer was preloaded with a spatial barcode to identify the location of the coordinates on the glass slide. Upon attaching the tissue to a slide and disrupting the cell membrane, mRNA in the tissue was captured by nearby oligo dT primers. Adding reverse transcriptase to synthesize cDNA makes it possible to synthesize a gene library that reflects its localization. Because the determined sequence contains the coordinate information, the detailed expression information of the coordinates can be obtained. The 10x Genomics company acquired, developed, and improved this ST technology to 55 μm/diameter resolution and commercialized it under the name “10x Visium” at the end of 2018. Visium results for breast cancer. Hematoxylin and eosin staining (left). Three cancer cell populations, classified via non-hierarchical K-means clustering (*k* = 9), are shown in different colors (middle panel). Four non-cancerous areas are shown to represent gene expression reflecting the microenvironments of the corresponding cancer cells (right panel) [54] (**e**). *PL* photocleavable linker, *CPP* cell-penetrating peptide, *PLP* padlock probe, *RCA* rolling circle amplification, *SBL* sequencing-by-ligation, *RT* Reverse transcription

Next, we profiled the spatial gene expression of breast cancer specimens via STseq using the Visium platform of 10x Genomics (Pleasanton, CA, USA), which is a commercial version of ISC-based ST analysis [54]. Figure 4 shows the Visium results. Clustering of expression information by the K-means method showed that cancer cells were classified into clusters 1–3, confirming the spatial heterogeneity of breast cancer cells. The cancer microenvironment is classified into four clusters. Interestingly, depending on the spatial differences in cancers, the microenvironment also differed. Even among morphologically identical cancer cells, there is heterogeneity in their expression profiles, suggesting that each cancer cell forms its own microenvironment.

### Cutting-edge Tools of STseq informatic analysis

**Fig. 5:**
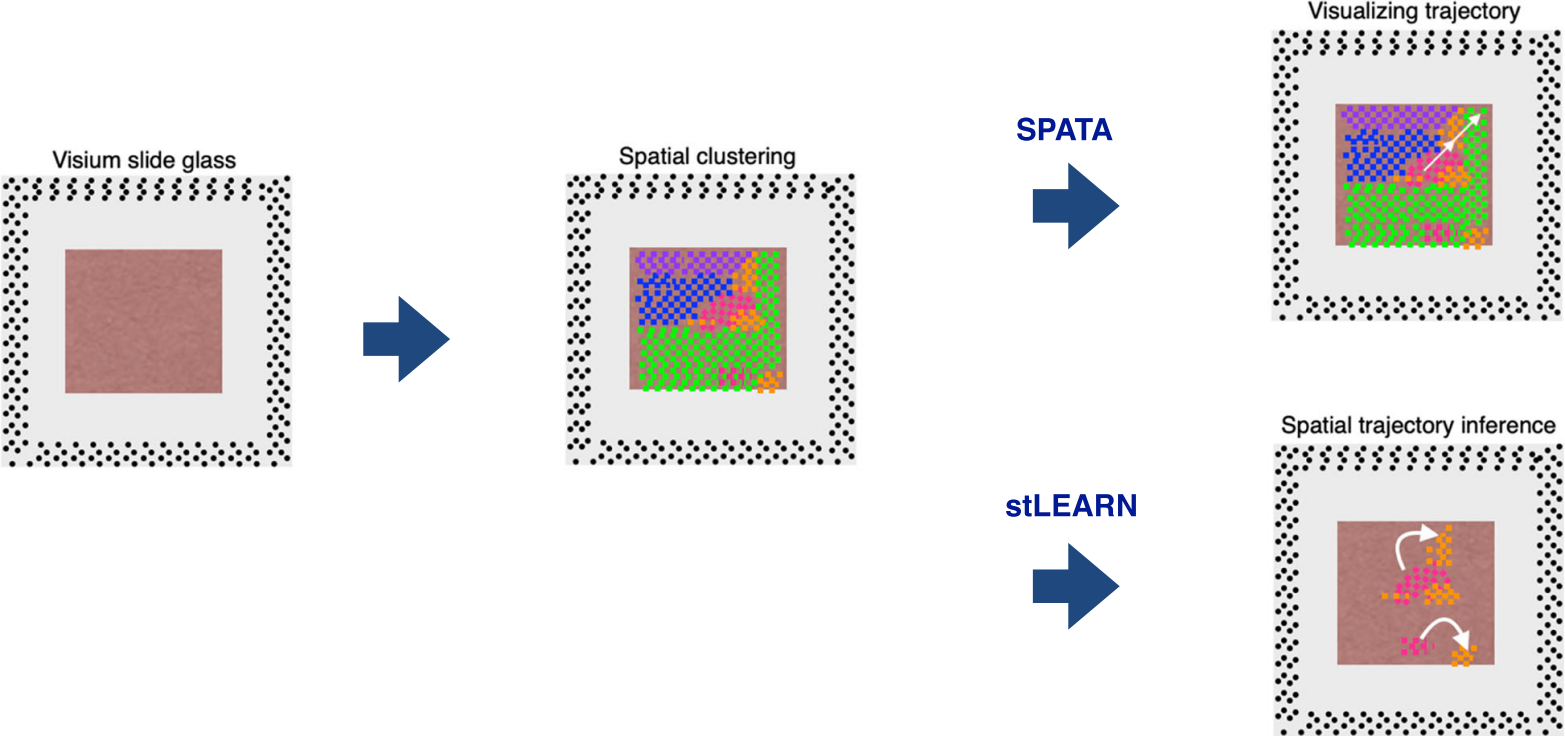
Tools for Spatial transcriptome analysis. Overview of analysis using SPATA and stLEARN. We can visualize trajectory by SPATA and can estimate spatial trajectory inference by stLEARN

As the experimental instrument technology developed, bioinformatic tools for spatial transcriptome are up coming. We introduce the paper using two different tools for STseq. Zhang et al. revealed the spatial trajectory of NSCLC samples from the data of 4 LUAD and 8 LUSC, lung squamous carcinoma using Visium dataset [55]. In addition to reveal the heterogeneity using Seurat [56], they conducted analysis using spatial-pseudo-time and revealed the spatial evolutional trajectory. First, to analyze transient gene expression along spatial trajectory, they used “SPATA,” a framework for spatial transcriptome analysis, [57]. It can analyze DEGs in spatial context. With SPATA analysis, they showed the invasion direction and continuous features (Fig. 5). They also used another tool called “stLEARN” to analyzed pseudo-time trajectory. With stLEARN, we can reveal the connections within subclusters (Fig. 5) [58]. From the result, they showed possible spatial evolution in NSCLC. Though the tool for STseq is still limited, we believe we can get much detailed biological mechanism using those technologies.

## Conclusions

We first reviewed a recent approach to better understand the molecular mechanisms underlying drug resistance. We introduced our recent single-cell analysis of osimertinib-resistant lung cancer cells. In the latter section, we reviewed the currently available ST analysis methods.

Single-cell analysis technologies allow for a bottom-up view of the organs and tissues from the perspective of cells. As a result, we can define various aspects, such as development, cancer heterogeneity, and infectious diseases, with a new and entirely different view. scRNA-seq revealed intratumor and intertumor heterogeneity. Recently, an increasing number of clinical samples, including anticancer therapy-resistant tumors, have been analyzed in combination with other single-cell level omics technologies. These single-cell multiomics analyses are expected to provide new findings.

Conversely, once the cellular architecture is destroyed during single-cell suspension preparations, the intuitive information viewed top-down is lost. STseq is a powerful method that can provide gene expression information without compromising the positional relationship between cells. Using 10x Genomics’ Visium, which is already commercially available, it is now possible to measure gene expression comprehensively and quantitatively with relative ease. However, the resolution of this method is insufficient.

The spatial concept within a cell is important to delve into the microenvironment within individual cells. It is possible to estimate the critical information related to the actual biological implications from the intracellular localization of the RNA molecule. Although ISS using PLPs, such as FishSeq and HybISS, may identify the genes at the subcellular level, the number of genes to be identified is limited due to the large size of RCPs and the short sequences to be decoded. In the future, it will be necessary to use both technologies in combination for analysis. We expect rapid progression of single-cell spatial omics technology that can capture time and space.

## Data Availability

Not applicable.

## Abbreviations

ChIP-seq: Chromatin immunoprecipitation sequencing
WES: Whole-exome sequencing
WGS: Whole-genome sequencing
scRNA-seq: Single-cell RNA sequencing
SAKE: Single-cell RNA-seq analysis and K/clustering evaluation
BRAF-I: BRAF inhibitor
scATAC-seq: Single-cell assay for transposase-accessible chromatin using sequencing
NSCLC: Non-small cell lung cancers
EGFR-TKI: Epidermal growth factor receptor tyrosine kinase inhibitor
GEX: Gene expression
UMI: Unique molecular identifier
CyTOF: Cytometry by time of flight
